# Childhood urbanicity interacts with polygenic risk for depression to affect stress-related medial prefrontal function

**DOI:** 10.1038/s41398-021-01650-x

**Published:** 2021-10-12

**Authors:** Xiao Zhang, Hao Yan, Hao Yu, Xin Zhao, Shefali Shah, Zheng Dong, Guang Yang, Xiaoxi Zhang, Timothy Muse, Jing Li, Sisi Jiang, Jinmin Liao, Yuyanan Zhang, Qiang Chen, Daniel R. Weinberger, Weihua Yue, Dai Zhang, Hao Yang Tan

**Affiliations:** 1 Peking University Sixth Hospital/Institute of Mental Health, 100191 Beijing, China; 2 NHC Key Laboratory of Mental Health (Peking University), National Clinical Research Center for Mental Disorders (Peking University Sixth Hospital), 100191 Beijing, China; 3 Department of Psychiatry, Jining Medical University, 272067 Jining, Shandong China; 4 Lieber Institute for Brain Development, Baltimore, MD 21205 USA; 5 Department of Psychiatry and Behavioral Sciences, Johns Hopkins University School of Medicine, Baltimore, MD 21205 USA; 6 Department of Neuroscience, Johns Hopkins School of Medicine, Baltimore, MD 21205 USA; 7 Department of Genetic Medicine, Johns Hopkins School of Medicine, Baltimore, MD 21205 USA; 8 PKU-IDG/McGovern Institute for Brain Research, Peking University, 100871 Beijing, China; 9 Tsinghua University-Peking University Joint Center for Life Sciences, 100871 Beijing, China

**Keywords:** Clinical genetics, Learning and memory, Clinical genetics, Depression

## Abstract

Urbanization is increasing globally, and is associated with stress and increased mental health risks, including for depression. However, it remains unclear, especially at the level of brain function, how urbanicity, social threat stressors, and psychiatric risk may be linked. Here, we aim to define the structural and functional MRI neural correlates of social stress, childhood urbanicity, and their putative mechanistic relevance to depressive illness risk, in terms of behavioral traits and genetics. We studied a sample of healthy adults with divergent urban and rural childhoods. We examined childhood urbanicity effects on brain structure as suggested by MRI, and its functional relevance to depression risk, through interactions between urbanicity and trait anxiety-depression, as well as between urbanicity and polygenic risk for depression, during stress-related medial prefrontal cortex (mPFC) engagement. Subjects with divergent rural and urban childhoods were similar in adult socioeconomic status and were genetically homogeneous. Urban childhood was associated with relatively reduced mPFC gray matter volumes as suggested by MRI. MPFC engagement under social status threat correlated with the higher trait anxiety-depression in subjects with urban childhoods, but not in their rural counterparts, implicating an exaggerated physiological response to the threat context with urbanicity, in association with behavioral risk for depression. Stress-associated mPFC engagement also interacted with polygenic risk for depression, significantly predicting a differential mPFC response in individuals with urban but not rural childhoods. Developmental urbanicity, therefore, appears to interact with genetic and behavioral risk for depression on the mPFC neural response to a threat context.

## Introduction

The world has been rapidly urbanizing, and in recent years especially so in Asia, bringing significant economic, social and environmental changes to traditional agrarian societies. Urban birth and childhoods have been associated with risk for schizophrenia [1, 2], autism spectrum disorders [3], substance dependence [4], as well as mood and anxiety disorders [5–8], but the mechanisms are not well understood. There are cultural and geographic variations in the social, economic and physical environments, which Nuance how urbanicity may influence neuropsychiatric risk. For example, urban living appears associated with chronic stress, depression [8] and psychiatric risk [9–12], from a number of studies in European-ancestry samples. However, there are recent suggestions that urbanicity risks, at least for depression, may diverge based on cultural-ethnic factors, which may differently modulate social support, the experience of social stressors, and subsequent depression risk [13]. It would therefore be important to account for ethnic-cultural and socioeconomic contexts, in defining the interacting environmental, and indeed potential genetic mechanisms of psychiatric risk.

Here, we studied a relatively socioeconomically and genetically homogeneous adult sample with diverse urban and rural childhoods stemming from recent decades of large-scale urbanization and transformation from a primarily agrarian to an industrial economy in China. As recently as the mid-1980s, some 63% of the Chinese population was engaged in rural, small-scale farming, but industrialization and urbanization has since rapidly accelerated, and China’s urban population has increased at a historic rate [14]. We leveraged this natural experimental context to examine how differing childhoods in urban and rural environments may influence adult brain structure measured with MRI and stress-related prefrontal cortical function in relation to depression risk, as indexed by trait anxiety-depression and polygenic risk for depression.

The prefrontal cortex and social evaluative stress have been implicated as possible targets of urban stress-related exposures and psychiatric risk [9–11]. Studies of medial prefrontal cortex (mPFC) function suggest that this region is critical for incorporating information on social hierarchies in affective regulation [15]. Medial PFC updates knowledge about one’s own social position [16], or self-estimates of performance [17], compared to that of others, and is critical in navigating complex social relationships and associated stresses. Medial PFC gray matter volume measured on MRI has been associated with strength of interpersonal relationships, social network size and resilience to stress [18]. Reduced mPFC gray matter volumes, and altered physiological activation have also been implicated in depression, and in trait anxiety-depression associated with risk for depression [19–22]. It is, however, less clear whether, and to what extent, these stress-related mPFC targets may be influenced by differences in childhood urbanicity, and if these environmental effects interact with genetic mechanisms associated with illness.

Indeed, if urban birth and childhoods influenced depression-related mPFC dysfunction through increased sensitivity to social stressors [9, 11], we might expect that these environmental effects would result in structural, and functional MRI changes in mPFC associated with states, as well as behavioral traits, related to interpersonal stress and depression. Trait anxiety-depression, while less studied in this neuroimaging context (but see Lederbogen et al. [9]), is a risk factor for depressive disorders and associated with underling genetic risk for depression [19–23]. We examined these hypotheses in a sample of healthy adult individuals living in Beijing, who experienced divergent rural or urban birth and childhood environments during some of history’s most rapid and widespread urbanization in the recent decades of China’s urban development [24]. Notably, it has been difficult in other study contexts to disentangle socioeconomic factors, and gene–environment correlations (e.g., more genetically susceptible individuals living in deprived environments). This complicates defining if, and how the childhood environment influences (i.e., interacts with) genetic mechanisms of disease. We aim herein to better control for and achieve relative independence of the environmental factors from genetic factors, i.e., the absence of gene–environment correlations. This should be facilitated by the unique scale of recent Chinese urbanization, where the population achieved levels of urbanization in 3–4 recent decades, that took Western countries some two centuries [24]. We posit this widespread urbanization should be less selective of genotype correlations with environment. Another feature of our study population is the relative homogeneity in current socio-economic status and genetic ancestry, which presents an opportunity to address questions about the childhood environment with better control for current life conditions.

Based on previous work [25, 26], we designed a working memory (WM) task under varying social status threat contexts. Because the mPFC is implicated in suppressing self-referential thoughts, especially of social stress in order to execute cognitive tasks such as WM [19, 27–30], we predicted that individuals with urban birth and childhoods, and particularly those with higher trait anxiety-depression, might have more aberrant neural threat responses at mPFC during this paradigm. Critically, to define the extent to which urban environments affect genetic aspects of depression risk, we then examined potential gene–environment interactions through which childhood urbanicity may influence stress-related prefrontal responses underlying polygenic risk for major depressive disorder [31].

## Methods

### Participants

Five hundred and twenty-two healthy adults were initially recruited from the local community in Beijing and 490 were included in the current study. (See Supplementary Methods for details of inclusion criteria, and clinical evaluation). Written informed consent was obtained from all participants. Of note in this report on social threat and trait anxiety-depression in brain, we used a validated Chinese translation of the Eysenck Personality Questionnaire to study trait anxiety-depression [32, 33]. The study was approved by the Institutional Review Boards of the Peking University Sixth Hospital and the Johns Hopkins University School of Medicine.

To determine urbanicity, subjects provided residence details from birth to present. We defined rural areas as agricultural regions with population typically <10,000; urban areas were defined as cities with populations typically more than 100,000 to well over several million. Here, we stratified subjects into an urban group, who had lived in cities since before they were age 12, and a rural group who were born in rural environments and only moved to cities after age 12 and beyond. However, similar structural and functional MRI results were obtained if we increased the resolution in which the differing childhood environments were quantified by stratifying subjects into four groups, or if we used an urbanicity score from previous studies [2, 9] (See Supplementary Methods).

### Structural MRI data acquisition and analysis

All subjects were scanned on a 3T GE Discovery MR750 scanner at the Center for MRI Research, Peking University (see Supplementary Methods for detailed acquisition and analyses). Voxel-based morphometry was performed using SPM8 (http://www.fil.ion.ucl.ac.uk/spm), DPABI [34], and DARTEL [35] to examine urbanicity effects. Significant effects survived *p* < 0.05 voxel-wise whole-brain family-wise error (FWE) correction for multiple comparisons.

### Functional MRI acquisition, task, and analysis

**Fig. 1:**
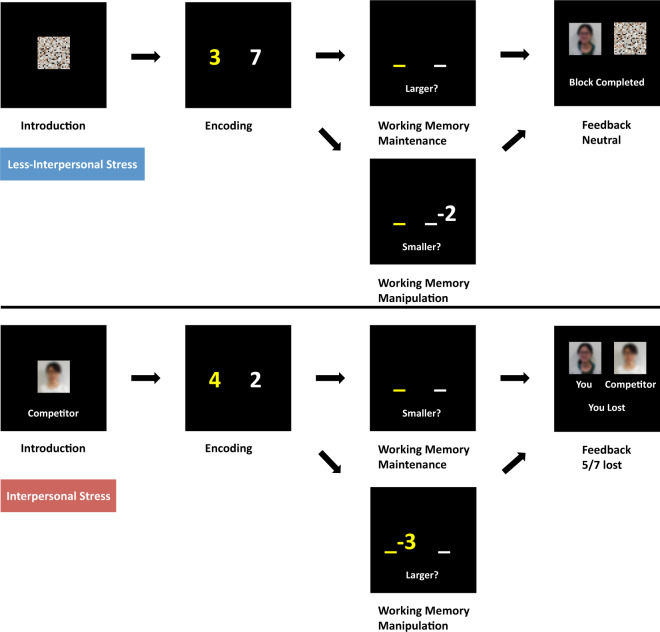
Working memory paradigm incorporating interpersonal threat stress. In the stressed component, subjects were led to believe they were playing against a “competitor” of the similar age and gender, and were judged as winning or losing based on their speed and accuracy, which subsequently resulted in ~70% loss feedback. In the less stressed blocks, there was no competitor and subjects received neutral feedback. In all the working memory manipulation and maintenance tasks, an array of two number digits was encoded and held in working memory over 3–4 s. In working memory maintenance, subjects responded to which of the two maintained digits was larger or smaller as indicated. In working memory manipulation, subjects performed subtraction on one of the numbers held in working memory, followed by a response as to which result was larger or smaller as indicated. Subjects performed two runs counterbalanced for trial and stimuli presentation order over ~20 min.

We adapted a “number working memory task” based on previous work [25, 26], and included social status threat stressors in half the trials in an event-related block design (Fig. 1). Subjects engaged counter-balanced “less stressed” and “stressed” numerical WM maintenance and manipulation trials. Under stress, they performed against a similar age and gender “competitor”, and subsequently received more negative (~70%) than positive feedback about their relative performance. In less stressed trials, they engaged without a “competitor” and received neutral feedback. (See Supplementary Methods for detailed task parameters.)

The functional MRI data were preprocessed and quality-controlled as previously [25, 26], and 394 subjects were included (See Supplementary Methods). To limit multiple-testing and to specifically examine the stress-related function of mPFC, we created functional regions-of-interest (ROIs) around the highest activation peaks in the left and right mPFC implicated in the structural MRI study (see “Results” section). These functional ROIs were defined independently of subsequent dependent variables of interest (urbanicity, trait anxiety, or polygenic risk for depression). In these ROIs, we then examined the hypotheses that depression risk manifested through trait anxiety-depression or polygenic risk for depression are implicated in the expression of states of interpersonal stress at mPFC [22], and how these may be modulated by childhood urbanicity [9]. Specifically, in each sub-sample with urban or rural childhoods, we examined the extent to which social threat-related engagement at mPFC during WM manipulation (or maintenance) might correlate with trait anxiety-depression, and their potential interaction. We considered significant effects as surviving whole-brain *p* < 0.001 uncorrected and *p* < 0.05 voxel-wise FWE corrected for multiple comparisons within the left (542 voxels) or right (722 voxels) mPFC functional ROI.

To define the extent to which childhood environments affect genetic aspects of depression risk, we examined the extent childhood urbanicity and polygenic risk for depression [31] may influence mPFC function associated with social status threat. Here, we randomly divided our functional MRI dataset into two sub-samples (discovery sample *N* = 200, and replication sample *N* = 194) of approximately equal numbers of individuals with urban or rural childhoods. In the discovery sample, at the same two mPFC ROIs, we examined the extent to which social threat-related engagement at mPFC during WM manipulation (or maintenance) might correlate with polygenic risk for depression in each urban or rural group, and their potential interaction. We considered significant effects as surviving whole-brain *p* < 0.001 uncorrected and *p* < 0.05 voxel-wise FWE corrected for multiple comparisons within the mPFC functional ROIs, unless otherwise stated. We examined for similar effects in the replication sample, and in the combined sample. To the extent childhood urbanicity interacted with genetic risk of depression to affect stress-related mPFC function, we further examined if this interaction was relatively specific to childhood urbanicity and stress-related depressive disorders, or if similar interaction effects could also be driven by childhood or current socioeconomic status, and genetic mechanisms typically less associated with stress-related psychiatric risk [36], such as height [37] and Alzheimer’s Disease [38].

### Genetic analyses

DNA collection and genome wide genotyping of the sample is described in Supplementary Methods. Principal component analysis (PCA) was performed to determine whether population stratification existed across our urban and rural samples. We evaluated the first 20 PCAs using a two-sample *t*-test with statistical significance set at *p* < 0.05 corrected for the number of independent components tested. The polygenic risk score for major depression disorder was calculated based on a recent genome-wide association study conducted by Psychiatric Genomics Consortium (PGC), which identified 44 independent loci [31] (see Supplementary Methods).

## Results

### Demographic and behavioral results

We studied 490 healthy adult subjects with differing birth and childhoods in urban (*N* = 249) and rural (*N* = 241) environments. Both groups had similar gender distribution, were all currently living in Beijing, and had achieved similar current educational and occupational levels (Table 1). Subjects with urban birth and childhoods were slightly younger and had higher parental education. Age and parental education effects on stress or cognitive-related dependent variables were then controlled for in subsequent behavioral and imaging analyses. Education and occupational levels remained similar across groups (Table 1). In terms of our hypotheses about social threat and trait anxiety-depression in relation to urbanicity, we found that trait anxiety-depression as measured on the Eysenck Personality Questionnaire Neuroticism score was higher in the urban group (*p* < 0.05, Table 1), but was independent of age or parental education. None of the subjects were diagnosed with a current or past mood or anxiety disorder. Psychoticism and extraversion scores did not differ across urbanicity. Further, in relation to childhood urbanicity, the subjects were genetically homogeneous with no significant differences across the first 20 principal components from genome-wide genotyping (Supplementary Fig. S1), or in polygenic risk for depression.

**Table 1:** Demographic and behavioral characteristics of subjects.

	Urban (SD)	Rural (SD)	*T*/*χ*^2^	*p*
Structural MRI study (*N* = 490)				
Gender (no. female/male)	131/118	119/122	0.512	ns
Age (years)	23.89 (3.98)	25.03 (3.45)	3.389	0.001
Education (years)	16.63 (2.32)	16.98 (2.62)	1.579	ns
Father’s education (years)	12.94 (3.57)	9.59 (3.29)	10.805	<0.001
Mother’s education (years)	12.42 (3.55)	7.88 (3.88)	13.515	<0.001
Current occupation (no.)			3.724	ns
Managers in public or private companies: Urban (5), Rural (5); Professionals and technicians: Urban (46), Rural (58); Clerks: Urban (12), Rural (10); Service Personnel: Urban (13), Rural (10); Students: Urban (164), Rural (145); Others: Urban (9), Rural (13).				
Functional MRI study (*N* = 394)				
Gender (female/male no.)	109/90	99/96	0.634	ns
Age (years)	23.69 (3.65)	24.96 (3.13)	3.707	<0.001
Education (years)	16.63 (2.32)	17.20 (2.53)	0.467	ns
EPQ neuroticism	7.60 (4.88)	6.65 (4.66)	1.968	0.049
Stress				
Accuracy in WM manipulation	0.86 (0.10)	0.86 (0.10)	0.471	ns
RT in WM manipulation (s)	1.43 (0.34)	1.54 (0.36)	0.431	ns
Accuracy in WM maintenance	0.92 (0.08)	0.92 (0.10)	0.903	ns
RT in WM maintenance (s)	1.04 (0.27)	1.11 (0.27)	0.336	ns
Less stress				
Accuracy in WM manipulation	0.85 (0.10)	0.86 (0.10)	0.650	ns
RT in WM manipulation (s)	1.48 (0.34)	1.58 (0.36)	0.409	ns
Accuracy in WM maintenance	0.86 (0.08)	0.86 (0.09)	0.442	ns
RT in WM maintenance (s)	1.10 (0.31)	1.19 (0.31)	0.517	ns

*EPQ* Eysenck personality questionnaire, *RT* reaction time, *WM* working memory.

We examined the extent to which the social threat stress contexts resulted in differential effects across WM tasks, to verify that the task paradigm indeed evoked predictable stress-related behavioral effects. During WM maintenance, trials with social threat were associated with relatively increased accuracy compared with less stress (*p* < 0.001, Table 1). There was, however, no stress-related effect on accuracy in WM manipulation, resulting in a significant task-by-stress interaction (*F* = 33.1, *p* < 0.001), (Supplementary Fig. S2 and Supplementary Table S1). During WM maintenance and manipulation trials, the evoked stress was associated with faster reaction times, also, with a task-by-stress interaction (*F* = 4.15, *p* < 0.05, Supplementary Table S1). These stress-evoked behavioral effects on our WM task are consistent with the well-established bias for better perseverative as opposed to flexible WM operations under stress and psychopathology [39–41].

Across the main urbanicity contrasts in WM maintenance or manipulation, there were, however, no behavioral differences in accuracy or reaction time, whether during the stressed, or less-stressed conditions (Table 1). There was no urbanicity-by-stress interaction on accuracy, or on reaction time during WM manipulation, or WM maintenance (Supplementary Table S1). Thus, the neural functional differences across urbanicity we subsequently detail below were unlikely driven by simple behavioral differences in WM, or associated educational and other confounders correlated with WM.

### Effects of urban vs. rural childhoods on gray matter volume in MRI

**Fig. 2:**
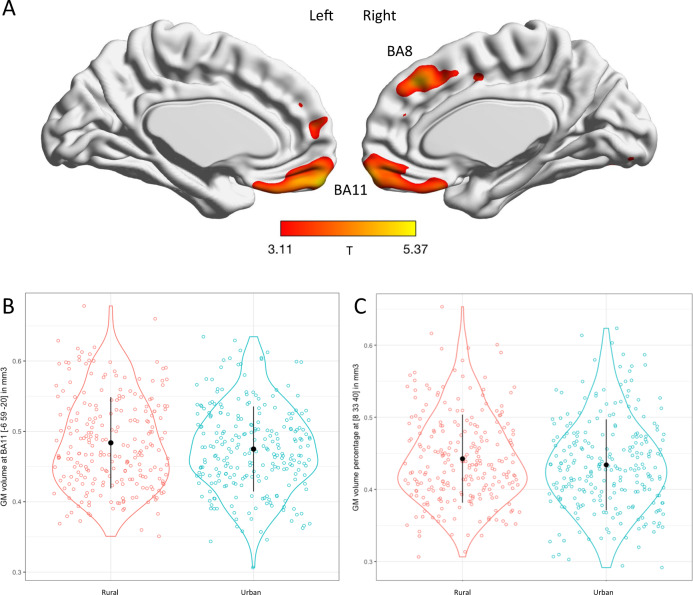
Childhood urbanicity effect on gray matter volume suggested by MRI (N = 490). **A** Brain map of the rural vs urban gray matter volume effects as suggested by MRI (shown at *p* < 0.001 uncorrected but peaks survived *p* < 0.05 whole-brain voxel-wise FWE corrected for multiple comparisons). **B** Plot of rural vs urban effects at Brodmann area 11 in medial prefrontal cortex (peak *x* = −6, *y* = 59, *z* = −20, *t* = 4.81, *p* < 0.05, whole brain voxel-wise FWE-corrected). **C** Plot of rural vs urban effects at Brodmann area 8 in mPFC (peak *x* = 8, *y* = 33, *z* = 40, *t* = 4.73, *p* < 0.05, whole-brain voxel-wise FWE-corrected).

Based on voxel-based morphometry in MRI, individuals with rural childhoods had relatively increased gray matter volume at the mPFC (*p* < 0.05, whole-brain voxel-wise FWE corrected for multiple comparisons, Fig. 2) in Brodmann area (BA) 11 (*x* = −6, *y* = 59, *z* = −20; *T* = 4.81) and BA8 (*x* = 8, *y* = 33, *z* = 40; *T* = 4.73). No other brain regions differed across urbanicity at these thresholds, or in the opposite contrast. As mPFC is critically engaged in processing social threat stress [15–17] potentially mediating urban-rural effects on risk for depression in brain, we then focused on mPFC as ROIs in subsequent functional MRI analyses.

### Effects of social status threat, trait anxiety and urbanicity on medial PFC function

During each of the WM maintenance and manipulation tasks, regions in the prefrontal, parietal and temporal cortices, and striatum, were robustly engaged, along with the well-established decreased engagement of the mPFC, the latter putatively associated with the degree to which self-representations needed to be suppressed to perform active cognitive tasks [19, 27–30] (*p* < 0.05 whole-brain voxel-wise FWE-corrected for multiple comparisons, Supplementary Fig. S3 and Supplementary Tables S2 and S3). Consistent with this formulation, under downgraded social status in the stressed vs the less stressed contexts, there was greater suppression of mPFC engagement during WM manipulation, as well as in WM maintenance (*p* < 0.05 whole-brain voxel-wise FWE-corrected for multiple comparisons, Supplementary Fig. S3 and Supplementary Table S4). The degree of medial PFC suppression was relatively “deleterious” and correlated with slower reaction times (*p* < 0.05 whole brain voxel-wise FWE corrected for multiple comparisons, Supplementary Fig. S4).

Stress-related functional ROIs in the left and right mPFC were subsequently defined, first, as the peaks sensitive to stress through the stress vs less stress contrast during WM manipulation, or maintenance, at *p* < 0.05 whole-brain voxel-wise FWE-corrected for multiple comparisons, that occurred within the BA11 region implicated in the structural MRI results (Fig. 2A). We then expanded these peaks to include regions that were similarly sensitive to stress at *p* < 0.05 whole-brain voxel-wise FWE-corrected for multiple comparisons within 30 mm of the highest peaks (Supplementary Fig. S3). These functional mPFC ROIs expanded the boundaries of the bilateral mPFC regions implicated in the structural results by margins of less than 8 mm—the smoothing kernel used in processing the functional data.

**Fig. 3:**
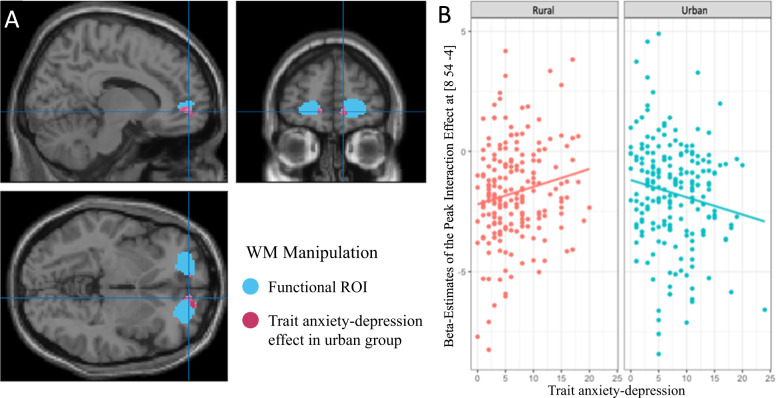
Effects of childhood urbanicity on medial PFC function during working memory manipulation under social threat stress. **A** Brain map showing the mPFC functional ROIs sensitive to stress vs less-stress contrasts (*p* < 0.05 voxel-wise FWE-corrected for multiple comparisons across the whole brain, blue), and peak correlation of mPFC suppression with increasing trait anxiety-depression during the WM manipulation task under social threat in individuals with urban childhoods (*N* = 199, *x* = 18, *y* = 60, *z* = 6, *T* = 3.26; *p* < 0.001, *p* < 0.05 voxel-wise FWE corrected for multiple comparisons within the mPFC ROI). **B** Plot of the interaction between rural and urban childhoods, and trait anxiety-depression on stress-related mPFC engagement (*N* = 394, *x* = 8, *y* = 54, *z* = −4, *T* = 3.37; *p* < 0.001 uncorrected, *p* < 0.05 voxel-wise FWE corrected for multiple comparisons within the mPFC ROI). In the group with more urban childhoods, increased trait anxiety-depression was associated with in a larger reduction in mPFC engagement during stress. In subjects with rural childhoods, however, this effect was less apparent.

As trait anxiety-depression is a behavioral risk factor for depression associated with the effects of social status threat [42, 43], and this trait was higher in urban childhoods in our study (above), we examined how stress-related mPFC function may interact with this trait in individuals with urban childhoods, and how this compared with rural childhoods at the mPFC. In individuals with urban childhoods, higher trait anxiety-depression was associated with greater suppression of mPFC engagement during WM manipulation under social threat stress (Fig. 3A, *x* = 18, *y* = 60, *z* = 8, *T* = 3.50, *p* < 0.001 uncorrected, *p* < 0.05 voxel-wise FWE corrected for multiple comparisons within the mPFC ROI). On the other hand, in those with rural childhoods, these effects were not significant. This resulted in a significant interaction at the mPFC in which subjects with urban childhoods and higher trait-anxiety-depression evinced greater suppression of self-related mPFC function [19, 27–30] to perform WM manipulation under social threat stress (Fig. 3B, *x* = 8, *y* = 54, *z* = −4, *T* = 3.37, *p* < 0.001 uncorrected, *p* < 0.05 voxel-wise FWE corrected for multiple comparisons within the mPFC ROI). These mPFC effects were independent of age or parental education. Effects during stress-related WM maintenance were not significant.

### Interaction between childhood urbanicity and polygenic risk of major depressive disorder on social threat-related medial PFC function

**Fig. 4:**
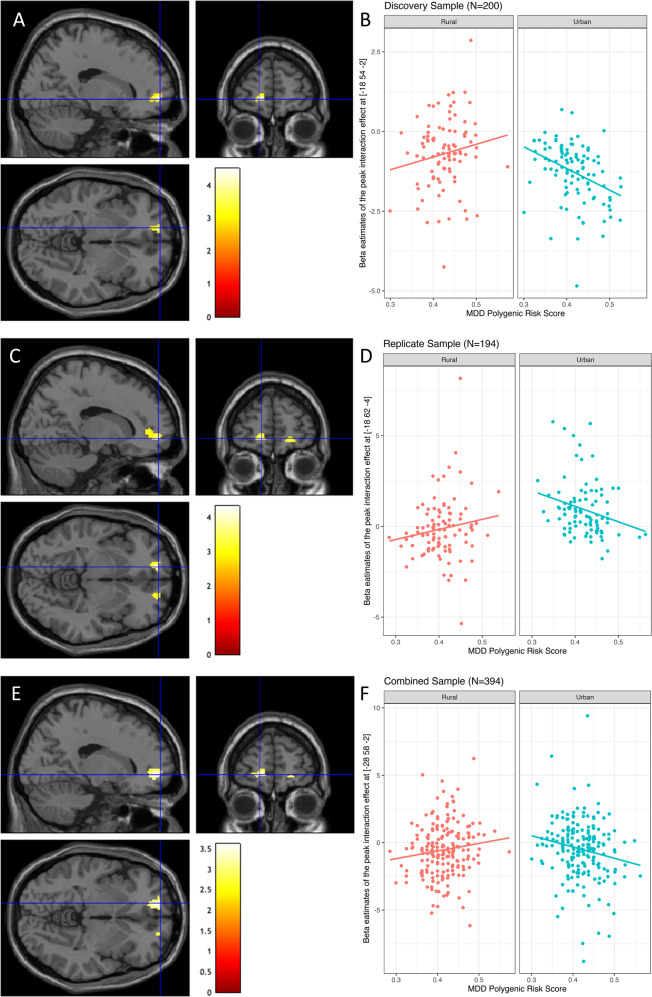
Effects of childhood urbanicity and polygenic risk for depression on stress-associated mPFC function during working memory manipulation. **A** In the discovery sample, peak effects in subjects with urban childhoods where polygenic risk for depression correlated with relatively deleterious reduced engagement of mPFC under stress vs. less stress (*p* < 0.001 uncorrected and *p* < 0.05 voxel-wise FWE corrected for multiple comparisons within the mPFC ROI). **B** Plot of the gene–environment interaction between rural or urban childhoods and polygenic risk for depression on stress-related mPFC engagement (*p* < 0.001 uncorrected and *p* < 0.05 voxel-wise FWE corrected for multiple comparisons within the mPFC ROI). In the group with urban childhoods, increased polygenic risk for depression resulted in disproportionately suppressed medial prefrontal cortex engagement during stress. This effect was less apparent in those with rural childhoods. **C** Similar effects were observed in the replication sample, where those with urban childhoods had more deleterious engagement of stress-related mPFC in relation to polygenic risk for depression (*p* < 0.001 uncorrected and *p* < 0.05 voxel-wise FWE corrected for multiple comparisons within the mPFC ROI). **D** These effects were absent in those with rural childhoods, resulting in a gene–environment interaction (*p* < 0.005 uncorrected). **E** In the combined sample, polygenic risk for depression was associated with deleterious stress related mPFC engagement (*p* < 0.001 uncorrected and *p* < 0.05 voxel-wise FWE corrected for multiple comparisons within the mPFC ROI) in those with urban childhoods. **F** The absence of these effects in those with rural childhoods resulted in a significant gene–environment interaction (*p* < 0.001 uncorrected and *p* < 0.05 voxel-wise FWE corrected for multiple comparisons within the mPFC ROI) in the combined sample.

In addition to trait anxiety-depression as a behavioral risk factor for depressive disorders, we examined polygenic risk for depression [31], and the extent to which childhood urbanicity and polygenic risk influenced mPFC function associated with social status threat. Here, we randomly divided our functional MRI dataset into two sub-samples of approximately equal numbers of individuals with urban or rural childhoods. In the discovery (*N* = 200 with 104 urban and 96 rural childhoods), replication (*N* = 194 with 99 urban and 95 rural childhoods) and combined samples, polygenic risk for depression did not differ across urban and rural childhoods, and was not associated with age, parental education, or with WM task accuracy or reaction time under stress, or less stress contexts. In the each sample, at the mPFC ROIs, we then examined the extent to which social stress-related engagement at mPFC might correlate with polygenic risk score in each urban or rural group, and their potential interaction. In the discovery sample, subjects with urban childhoods and higher polygenic risk for depression showed greater suppression of mPFC engagement under social threat stress relative to less stress during WM manipulation (Fig. 4A; *x* = −18, *y* = 54, z = −2, *T* = 4.49, *p* < 0.001 uncorrected, *p* < 0.05 voxel-wise FWE small-volume corrected for multiple comparisons within the mPFC ROI). These effects were not significant in subjects with rural childhoods, resulting in a significant gene-by-environment interaction at the mPFC (Fig. 4B; *x* = −18, *y* = 54 *z* = −2, *T* = 3.58, *p* < 0.001 uncorrected, *p* < 0.05 voxel-wise FWE corrected for multiple comparisons within the mPFC ROI). Effects in these mPFC ROIs during stress-related WM maintenance were not significant.

In the replication sample, subjects with urban childhoods also had more suppressed mPFC engagement during WM manipulation under social stress relative to less stress that correlated with polygenic risk for depression (Fig. 4C; *x* = −16, *y* = 56, *z* = −6, *T* = 4.36, *p* < 0.001 uncorrected, *p* < 0.05 voxel-wise FWE corrected for multiple comparisons in the mPFC ROI). These effects were not significant in subjects with rural childhoods, resulting again in significant a gene–environment interaction at the mPFC (Fig. 4D; *x* = −18, *y* = 62, *z* = −4, *T* = 2.69, *p* < 0.005). In the combined sample, the effect of stress-related mPFC engagement on polygenic risk for depression in the subjects with urban childhoods (Fig. 4E; *x* = −18, *y* = 58, *z* = −4, *T* = 3.61, *p* < 0.001 uncorrected, *p* < 0.05 voxel-wise FWE corrected for multiple comparisons within the mPFC ROI), but not in those with rural childhoods, resulted in a significant gene–environment interaction at the mPFC (Fig. 4F; *x* = −28 *y* = 58, *z* = −2, *T* = 3.23, *p* < 0.001 uncorrected, *p* < 0.05 voxel-wise FWE corrected for multiple comparisons within the mPFC ROI).

We then examined the extent to which the stress-related mPFC effect may be relatively specific to putative stress-related effects of urban childhoods and depression genetic risk. Childhood socioeconomic status as indexed by parental education (*p* > 0.11), or current socioeconomic status as indexed by occupation (*p* > 0.23) and education (*p* > 0.50), did not significantly interact with polygenic risk for depression to influence stress-related mPFC function. Polygenic risk scores of phenotypes putatively less strongly associated with stress or depression [36], in particular, height [37], and Alzheimer’s Disease [38], did not show significant interactions with childhood urbanicity in influencing stress-related mPFC function (*p* > 0.30, Supplementary Fig. S5).

## Discussion

In a relatively large sample of putatively normal subjects, we examined the effects of urban and rural birth and childhoods on mPFC brain structure as suggested by MRI, and on stress-related mPFC function associated with behavioral and genetic risk for depression. The sample is unique in having had similar current educational and occupational status, and genetic ancestry, but with divergent childhoods during China’s recent rapid urbanization. This study context allowed us to isolate the role of urban vs rural childhoods on brain development and function in relation to genetic risk for depression. Prior seminal work has highlighted the role of mPFC in mediating the effects of urbanicity and increased social stress sensitivity and trait anxiety-depression in European populations [9], though the association with illness-associated genetic risk has been less clear. We add to this earlier work, the finding that urban birth and childhoods affected the MRI correlates of structure and function of mPFC, wherein childhood urbanicity was associated with more “deleterious” stress-related mPFC function and higher trait anxiety-depression. Furthermore, the stress-related mPFC effects were correlated with polygenic risk for major depressive disorder in association with urban but not rural childhoods, in a significant gene–environment interaction. These interactions were not associated with childhood or adult socioeconomic differences. There were no genetic differences across urbanicity, and no apparent childhood urbanicity interactions at stress-related mPFC function with genetic risk for height and Alzheimer’s Disease—phenotypes putatively not genetically correlated with depression and stress-related psychiatric traits [36], and less strongly associated with chronic mild stress and urbanicity. These data potentially implicate childhood urbanicity in mediating the genetic risk mechanisms of depressive (and related psychiatric) illness through social stress-related mPFC function.

Our findings of putatively reduced mPFC gray matter volumes as measured with MRI in relation to urban birth and childhoods may implicate, though indirectly, increased adverse stress exposure. Medial PFC gray matter volume reductions in MRI have been observed in childhood adversity [44, 45], depression [21], post-traumatic stress disorder [46], and in relation to increased trait anxiety [22]. However, the underlying mechanisms by which urban birth and childhood is associated with prefrontal cortical variation is less understood. There is prior evidence that urban childhoods may be characterized by more stressful social environments, greater socioeconomic disparities and associated threats to social status [47–49], including in East Asia [50]. Parental stress may also affect prenatal and postnatal child development [51], and have been associated with increased trait anxiety-depression [52–54]. These risk traits could contribute to states of stress through tendencies to mis-appraise the social environment as threatening and coping resources as low [55–58]. Cognitive appraisal engages executive function and lateral and medial prefrontal cortex [59], also known to be sensitive to stress, at least in part through dopaminergic mechanisms [60]. Indeed, maladaptive self-referential ruminations characteristic of trait anxiety-depression [61] relate to mPFC function, which under active cognitive task demands is suppressed [19, 27–30]. These prior studies suggest that if indeed urban birth and childhoods affect depression risk and cortical function under stress, then urbanicity should affect depressive traits, and executive function under stress. These assumptions are consistent with our findings, where urban, but not rural childhoods potentiated relative mPFC dysfunction under social and cognitive stress during WM, in relation to higher trait anxiety-depression. As negative thoughts characteristic of trait anxiety-depression could be moderated by experience of nature through mPFC function [62], it may also be conceivable that rural environments could moderate the influence stress-related mPFC development and function [63, 64], consistent with our observations. Moreover, similar MRI results were obtained if we increased the resolution in which the differing childhood environments were quantified (See Supplementary Figs. S6–8).

To the extent that social stress-related mPFC vulnerabilities in urban childhoods affect illness-related genetic brain mechanisms, we found that these differing childhoods also interacted with the mPFC effects of genome-wide genetic risk for depression. The gene–environment interaction observed suggests that urban childhoods potentiate the effects of risk-associated genetic variation for depression at the level of stress-related mPFC biology. Previous enrichment analyses of the GWAS findings to bulk tissue mRNA-seq from the Genotype-Tissue Expression (GTEx) data [65] show that the most significant enrichments were at PFC and mPFC [31]. Our findings further suggest that specific stress-related aspects of urban birth and childhoods may affect genetic pathways to potentiate mPFC dysfunction under stress. The mPFC effects were apparently not driven by childhood urbanicity interactions with genetic factors less strongly related to stress and psychiatric risk, such as genetics associated with height, or Alzheimer’s disease [36]. On the other hand, while we have focused herein on polygenic risk for depression, this genetic risk is known to overlap with that for schizophrenia, mood and anxiety disorders, obsessive-compulsive disorder and attention deficit hyperactivity disorder [36], illnesses also linked to stress. Further defining the psychiatric disease-specific, and/or common genetic and environmental mechanisms in brain should be targets of future work. Our data are also limited to the mPFC effects associated with childhood urbanicity on brain structure in MRI, and accompanying hypotheses targeting mPFC in stress-related biology. The degree to which networks involving mPFC and beyond are implicated should also be considered in future. Nevertheless, our data suggest that threat-related stressors in the early urban environment may at least in part be implicated in depressive risk, and may be manifest through mPFC and stress-related cognitive functions.

That polygenic depression risk derived from European populations correlated with the mPFC biology of an East Asian sample supports the trans-ancestry importance of these large GWAS meta-analyses [31]. We suggest at least some of these shared genetic risks are relevant at the level of biologic phenotypes influenced by critical childhood environments. We posit it is possible to leverage these diverse data to improve understanding of gene–environmental mechanisms of illness [66]. Our work also extends evidence of overlapping neuropsychiatric GWAS effects across European and East Asian populations [67], including in GWAS of depression and major psychiatric disorders [68]. Nevertheless, we anticipate future work on more ancestry specific sets of genetic risk variation for depression, could more powerfully define specific subsets of genetic and environmental brain mechanisms in our study population.

Lastly, our data suggest there may be factors that allowed our sample of individuals with rural childhoods to do comparably well socioeconomically in Beijing as the more urban group. This may include advantageous mPFC function under stress, which was less associated, or possibly more resilient to genetic risk and to trait anxiety-depression, relative to the urban group with higher trait scores. These effects are unlikely to be confounded by genetic ancestry per se across urban–rural exposures. The more specific genetic and environmental factors in these urban and rural contexts, and how they impact larger-scale brain networks remain to be further defined. Neural circuitry engaging the mPFC, stress-related urban early life exposures, and interacting psychiatric risk genes would be initial targets for this future work.

## Supplementary information

Supplementary Materials
