# Supplementary material for: Childhood urbanicity interacts with polygenic risk for depression to affect stress-related medial prefrontal function
Source: Transl Psychiatry. 2021 Oct 12;11:522. doi: 10.1038/s41398-021-01650-x (PMC8511000; doi:10.1038/s41398-021-01650-x)
Supplement: Supplementary file 1 — Supplementary Materials [file 41398_2021_1650_MOESM1_ESM.pdf]

Supplementary Data

Supplementary Methods

Participants

This study was approved by the Institutional Review Boards of the Peking University Institute of Mental Health and the Johns Hopkins University School of Medicine. 522 healthy subjects were initially recruited from the local community in Beijing, and written informed consent was obtained from each subject. We recruited subjects by advertising the study using social media and flyers in the community. All participants were assessed by psychiatrists using the Structured Clinical Interview for DSM-IV-TR Axis I Disorders, Research Version, Non-patient Edition (SCID-I/NP).

Inclusion criteria were as follows: age 18 to 45 years; right handed; Chinese of Han ancestry; no history of psychiatric or neurological diseases and substance abuse or dependence; no history of loss of consciousness duration more than 5 minutes; and no structural abnormalities on subsequent MRI, read by trained radiologists. In this study, all subjects also had to be currently living in Beijing for at least one year, and provided residence details from birth to study enrollment. They had to have at least finished the national nine-year education program. We excluded 2 subjects with poor quality of structural MRI images, and 490 subjects were included in the subsequent structural MRI analyses.

To determine urbanicity, subjects provided residence details from birth to present. We defined rural areas as agricultural regions with population typically <10,000; urban areas were defined as cities with populations typically more than 100,000 to well over several million. In the main text, we stratified subjects into an urban group, who had lived in cities since before they were age 12, and a rural group who were born in rural environments and only moved to cities at or after age 12. However, similar structural and functional MRI results (See Supplementary Figures S6, S7 and S8) were obtained if we increased the resolution in which the differing childhood environments were quantified by stratifying subjects into 4 groups, or if we used an urbanicity score from previous studies [1,2]. In the former, the 4 groups were as follows: individuals who were born in and continue to live in cities (N=123), those who have lived in cities since before age 12 (N=126), those who lived in rural areas between birth and age 18 (N=113), and those lived in rural areas for >18 years since birth (N=128). In the case of the urbanicity score[1,2], this was defined according to population size as follows: population < 10000 = 1, less than 1,000,000 residents = 2; cities with more than 1,000,000 residents = 3; the category scores were then multiplied by the number of years spent in the location until age 15.

MRI Data Acquisition:

All subjects were scanned on a 3.0 T GE Discovery MR750 scanner at the Center for MRI Research, Peking University. The T1-weighted high-resolution structural image was acquired in a sagittal orientation using an axial 3D fast, spoiled gradient recalled (FSPGR) sequence with the following parameters: time repetition (TR) = 6.66 ms, time echo (TE) = 2.93 ms, field of view (FOV) = 256 × 256 mm^2, slice thickness/gap = 1.0/0 mm, acquisition voxel size=1 × 1 × 1mm^3, flip angle = 12°, 192 contiguous sagittal slices.

As for functional MRI, each echo-plannar image consisted of 33 (4.2 mm thick, 0 mm gap) axial slices covering the entire cerebrum and cerebellum (TR/TE = 2000/30 ms, flip angle = 90°, field of view = 22.4 cm, 64 x 64 matrix). Scanning parameters were selected to optimize the stability and quality of the BOLD signal with the exclusion of the first 4 images as dummy scans.

Structural MRI Data Analysis

Structural MRI Data Analysis involved the following steps: (1) Transforming structural images into NIFTI format. (2) Reorienting structural images iteratively so that the millimeter coordinates of the anterior commissure (AC) matched the origin. (3) T1-weighted MR images were segmented into grey matter, white matter, cerebrospinal fluid using "New Segment" in SPM8. (4) DARTEL was used to compute transformations from individual native space to Montreal Neurological Institute (MNI) space for registration, normalization, and modulation. (5) The segmented, normalized and modulated GM images were then smoothed with an 8-mm full width at half maximum isotropic Gaussian kernel.

To study the effect of relative urban-rural childhoods on brain structure, an absolute threshold of 0.2 was used to remove voxels of low intensity from the analysis and to prevent possible edge effects[3]. Voxel-based morphometry was then performed using SPM8 (http://www.fil.ion.ucl.ac.uk/spm), controlling for the effects of age, second polynomial of age, gender, education years, and total gray matter volume. Significant effects were those that survived a $p<0.05$ whole brain family-wise error (FWE) correction. While we cannot exclude the potential effects of head motion on measures of brain structure [4], we confirmed that there were no significant group differences in the six head motion dimensions from the functional MRI acquisition performed in the same MRI session.

Functional MRI Task and Analysis

We adapted an event-related "number working memory task" based on previous work[5,6]

(Figure 1). Subjects were trained outside the scanner for about 10 minutes. For working memory (WM), subjects encoded 2 integer numbers presented over 1s and retained in WM across a jittered interval of 3-5 seconds; in maintenance trials, subjects then responded to which of the two numbers was "larger" or "smaller" within 2s; in the manipulation trials, subjects had to perform a mental subtraction of 2 or 3 on one of the two numbers before the "larger" or "smaller" evaluation within 2s. There were 28 trials of WM manipulation and 28 trials of WM maintenance, half of which included competition and relatively more stress. Trials were embedded within equal numbers of competition or no-competition blocks, each comprising one WM maintenance and one WM manipulation trial, counterbalanced within 2 MRI runs, each about 10 minutes. Each block of competitive or non-competitive events was preceded by an initial instructional cue, whereby for competition, the participants were led to believe they were playing against a "competitor" of the same gender and of similar age, and after each WM trial, were given win or loss feedback. Here, subjects were given negative (loss) feedback approximately 70% of the time.

In the functional MRI data analysis, we excluded subjects with lower quality data according to similar criteria we have reported before[5,6], which were accuracy rate lower than 50 percent on WM maintenance or manipulation (n = 37), those with head motion greater than 2 mm translation or 2 degrees rotation (n = 43), and those with image artifacts or did not complete the task (n=16). Functional imaging analysis was performed using SPM12 (http://www.fil.ion.ucl.ac.uk/spm) and Matlab 2016b. Functional images for each subject was slice timing corrected, realigned to the first volume in the time series, and corrected for head motion. Images were then spatially normalized into standard stereotaxic space (Montreal Neurological Institute template) using a fourth degree B-spline interpolation. Spatial smoothing was applied with a Gaussian filter set at 8mm full-width at half-maximum. Each task-evoked stimulus was modeled as a separate delta function and convolved with a canonical hemodynamic response function, ratio normalized to the whole-brain global mean to control for systematic differences in global activity, and temporally filtered using a high-pass filter of 128s. Each task-evoked stimulus event was modeled for correctly performed trials. Incorrect responses and residual movement parameters were also modeled as regressors of no interest. In our study, planned contrasts of interest were brain activity at the maintenance or manipulation task phases under less stress, stress, and less stress vs stress. These contrasts were subsequently taken to a second-level analysis in which inter-subject variability was treated as a random effect.

In this paper, we focus on the stress-related medial prefrontal (mPFC) region-of-interest in bilateral BA11, implicated in the structural MRI results (Figure 2A), that were also sensitive to stress through the less stress vs stress contrast during WM manipulation, or maintenance, at the combined group level at $p<0.05$ whole-brain FWE-corrected for multiple comparisons. We then enlarged the ROIs by creating 30mm diameter spheres from the respective peak voxel in the stress-associated contrast at the left and right BA11. From these two orthogonally defined ROIs for each WM manipulation or maintenance task, we tested for

correlations of stress-associated mPFC function during WM manipulation, or maintenance, with trait anxiety, or polygenic risk for depression, in each group of individuals with urban or rural childhoods separately, as described in detail the Methods and Results. Statistical significance was set at $p<0.001$ uncorrected and $p<0.05$ small volume FWE corrected for multiple comparisons within the ROI. Where correlation results differed between urban and rural groups, we tested for interactions using a full model comprising the main and interaction effects, at the same statistical thresholds unless stated otherwise.

DNA Collection and Genotyping

Genomic DNA from the Beijing samples were extracted from peripheral blood using the QIAamp DNA Mini Kit (QIAGEN). Genotyping of samples was conducted using Illumina Human Omni ZhongHua BeadChips, designed for the Chinese population. Normalized bead intensity data obtained for each sample were loaded into Illumina BeadStudio software, which converted fluorescence intensities into SNP genotypes. Samples were excluded (N=15) according to the following quality-control criteria: (1) genotype call rate of <95%, (2) gender discordance, (3) first- or second-degree relatedness, or (4) the genetic outliers. SNPs were excluded using the following criteria: (1) minor allele frequency (MAF) <0.01, (2) genotype call rate of <95%, (3) P values for Hardy-Weinberg equilibrium < 1e-5. Principal Component Analysis (PCA) was performed to identify genetic outliers and determine whether population stratification existed between our urban and rural samples, using EIGENSTRAT (http://genetics.med.harvard.edu/reich/Reich_Lab/Software.html). We compared the first 20 PCAs among the two urbanicity groups using a two-sample t-test with statistical significance set at $p<0.05$ corrected for the number of independent components tested.

The polygenic risk score for major depression disorder was calculated based upon a recent genome-wide association study conducted by Psychiatric Genomics Consortium (PGC), which identified 44 independent loci[7]. Using PLINK v1.07 software[8], we calculated the depression polygenic risk score based on the 44 lead SNPs of each susceptibility loci. Each SNP was weighted by the effect size from the GWAS, and weighted sums were used to compute the GRS score. Genotype imputation was carried out via the pre-phasing/imputation stepwise approach[9]. Genotypes were first phased using SHAPEIT (v2.r727)[10], and imputation was then performed over each 3 Mb interval centered on all index SNPs using IMPUTE (v2.3.0)[9] software. Haplotypes derived from Phase I of the 1000 Genomes Project (release v3) were used as reference data. Polygenic risk scores were similarly derived from recent large cohorts reporting for height[11] and Alzheimer's Disease[12].

Supplementary Figures:

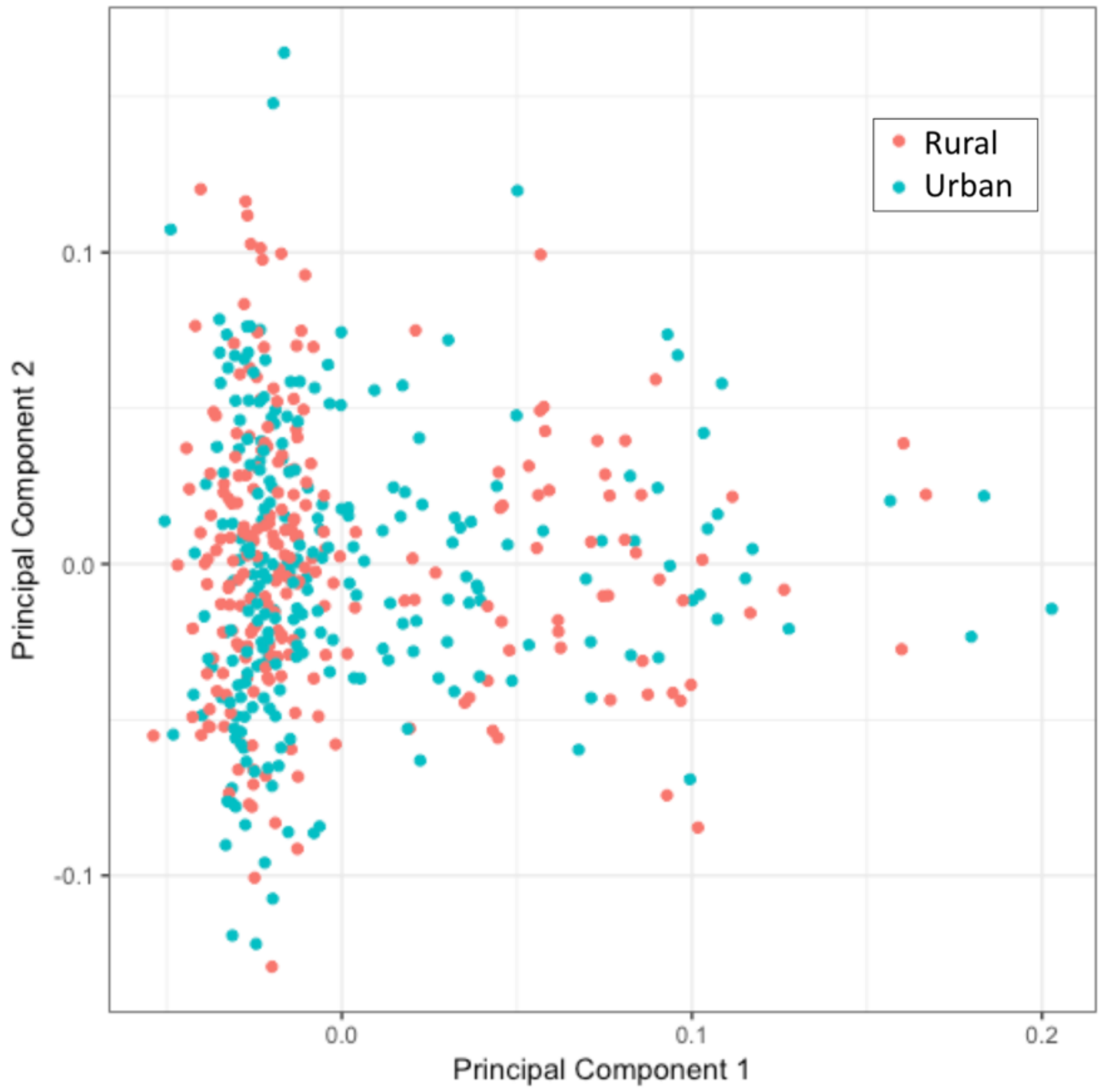

Fig. S1. ***Principal components analysis on whole genome genotyping.*** The plot shows overlapping first and second principal components across rural (red) and urban (blue) groups. We also compared the first 20 principal components across rural and urban groups, and there were no significant differences.

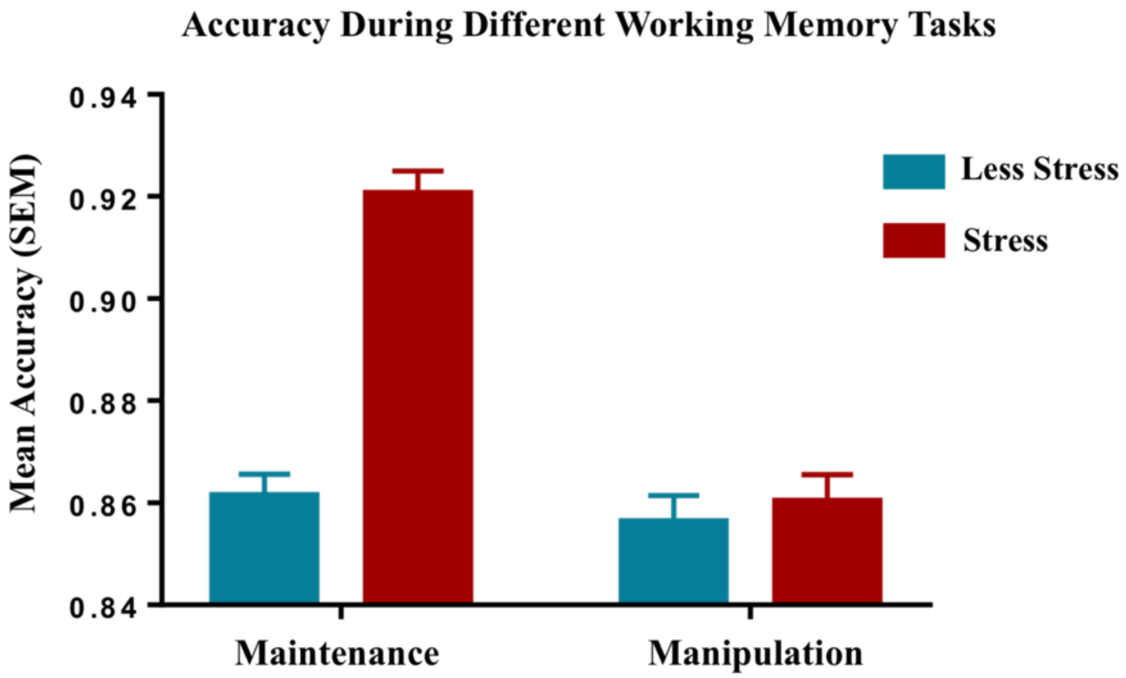

Fig. S2. ***Accuracy during WM maintenance and manipulation in the whole sample (N=394).*** During WM maintenance, trials with interpersonal stress were associated with relatively increased accuracy ($p<0.001$). This effect was not seen during WM manipulation, resulting in a significant task by stress interaction ($p<0.001$) consistent with a well-established bias for perseverative as opposed to flexible WM operations under stress[13,14].

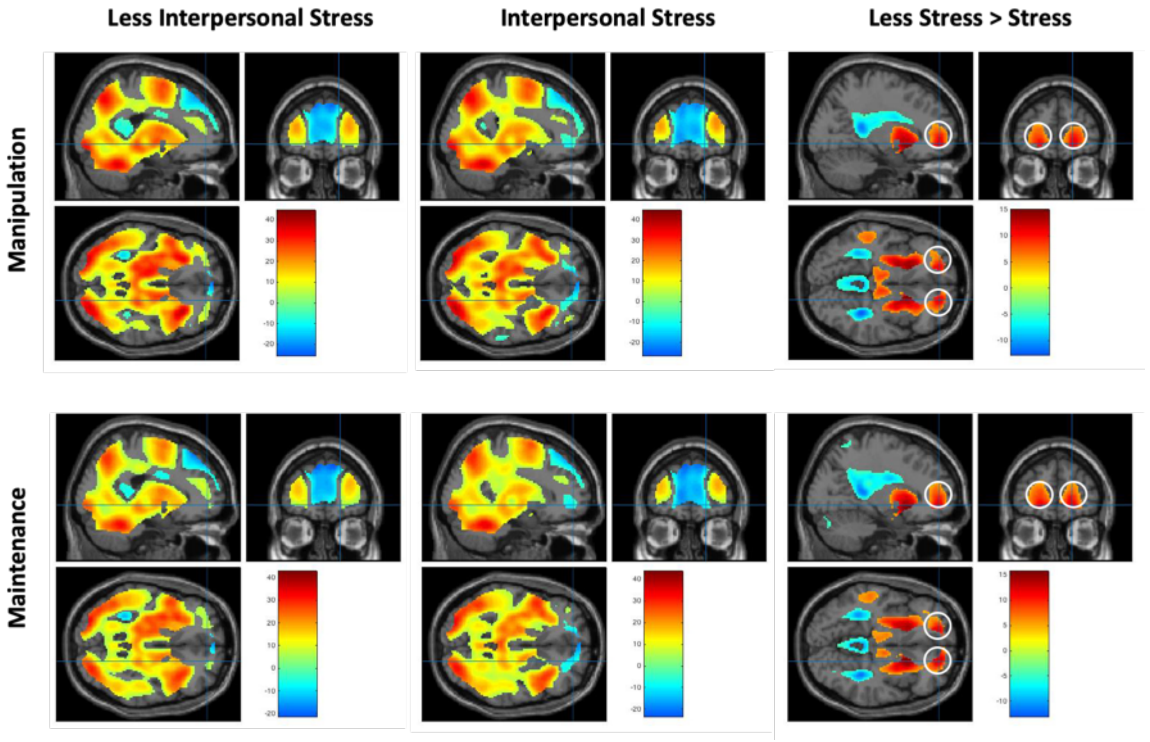

Fig. S3. ***Brain activation under working memory manipulation and maintenance tasks under differing social threat stress conditions.*** One-sample t-tests of working memory (WM) task across the entire sample (N=394, controlling for age; $p < 0.05$, voxel-wise whole brain FWE corrected, cluster size > 100; peaks detailed in Supplementary Tables S2-S4) under differing stress and working memory manipulation and maintenance conditions. Medial prefrontal cortex (mPFC), amongst other brain regions, was less engaged under stress. At the highest peak stress-related effect during WM manipulation, or maintenance at $p<0.05$ voxel-wise whole-brain FWE-corrected, that is within the left and right mPFC implicated in the structural results (Fig 2), we defined 30mm diameter functional ROIs (white circles) intersected by voxels sensitive to stress through the orthogonal stress vs less stress contrast at $p<0.05$ voxel-wise whole-brain FWE-corrected. Manipulation: Right mPFC peak (ROI center), x=20, y=52, z=-6, T=11.23, 722 voxels; left peak, x=-18, y=50, z=-8, T=10.65, 542 voxels. Maintenance: Right peak, x=18, y=52, z=-6, T=12.24, 743 voxels; left peak, x=-18, y=50, z=-6, T=11.96, 725 voxels.

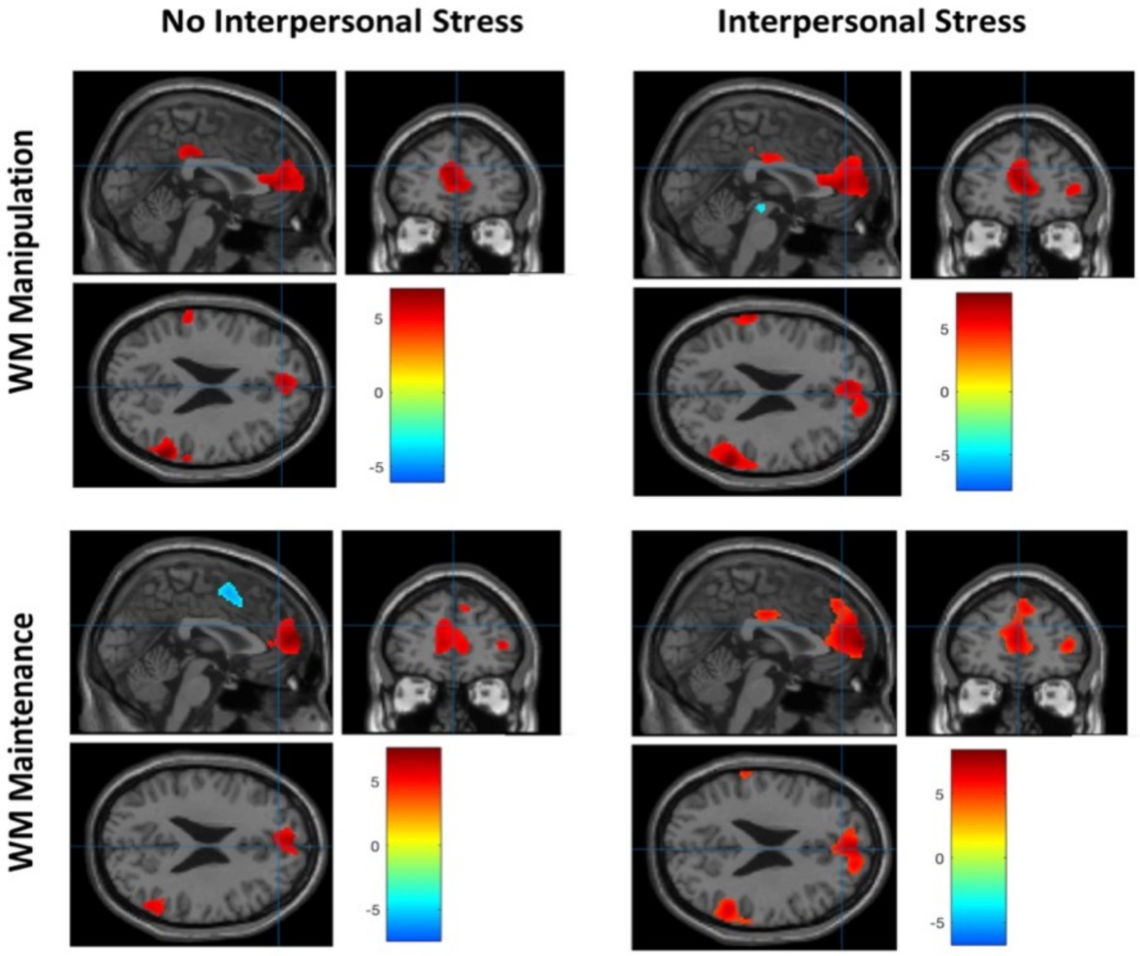

Fig. S4. ***Effects of speed of processing during WM brain activation.*** Correlation analysis of speed of processing (reciprocal of reaction time) and brain activation across WM manipulation and WM maintenance (N=394, controlling for age, $p < 0.05$ voxel-wise whole brain FWE corrected, cluster size > 100). Medial prefrontal cortex had enhanced engagement (less suppression) in relation to faster processing across WM tasks. Conversely, greater 'deleterious' suppression of mPFC function was associated with slower processing across WM tasks.

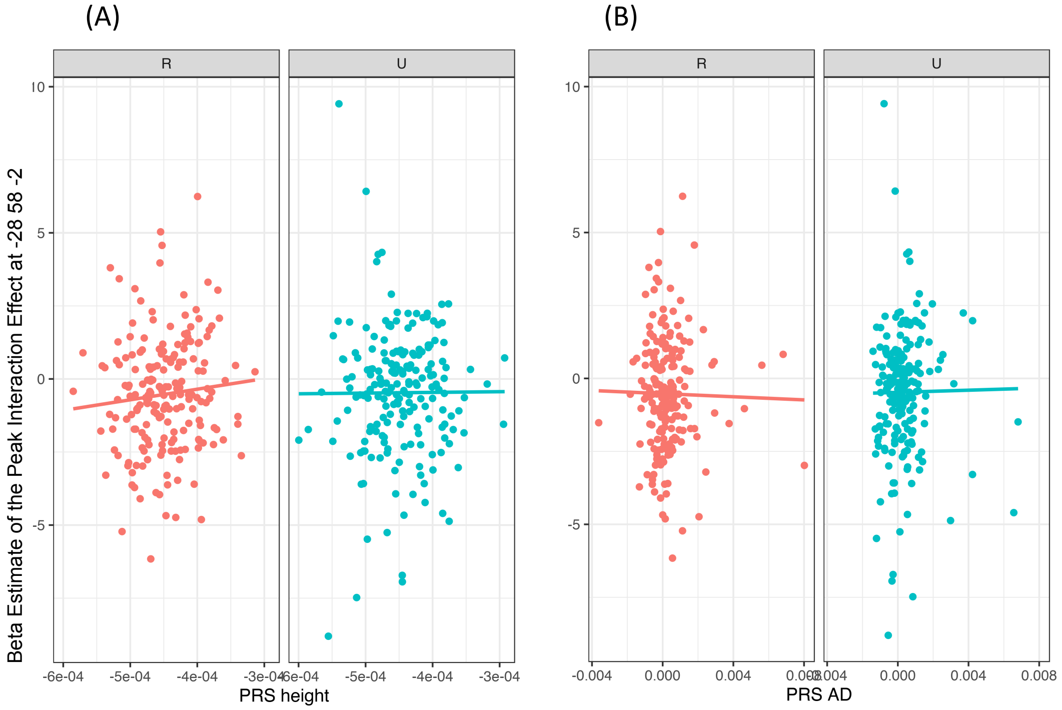

Fig. S5: ***No significant effects of genetic risk of phenotypes putatively less associated with stress and childhood urbanicity on medial PFC function.*** (A) At stress-related medial PFC function previously found to be associated with childhood urbanicity and genetic risk for depression (Fig 4E), we examined the extent to which this effect may be driven by genetic risk less associated with stress and childhood urbanicity. There was no significant interaction effect across polygenic risk for height and childhood urbanicity (red: urban childhood, blue: rural childhood, T=0.76, p>0.3) at stress-related medial PFC function. (B) There was no significant interaction effect across polygenic risk for Alzheimer's Disease and childhood urbanicity (T=0.26, p>0.3) at stress-related medial PFC function.

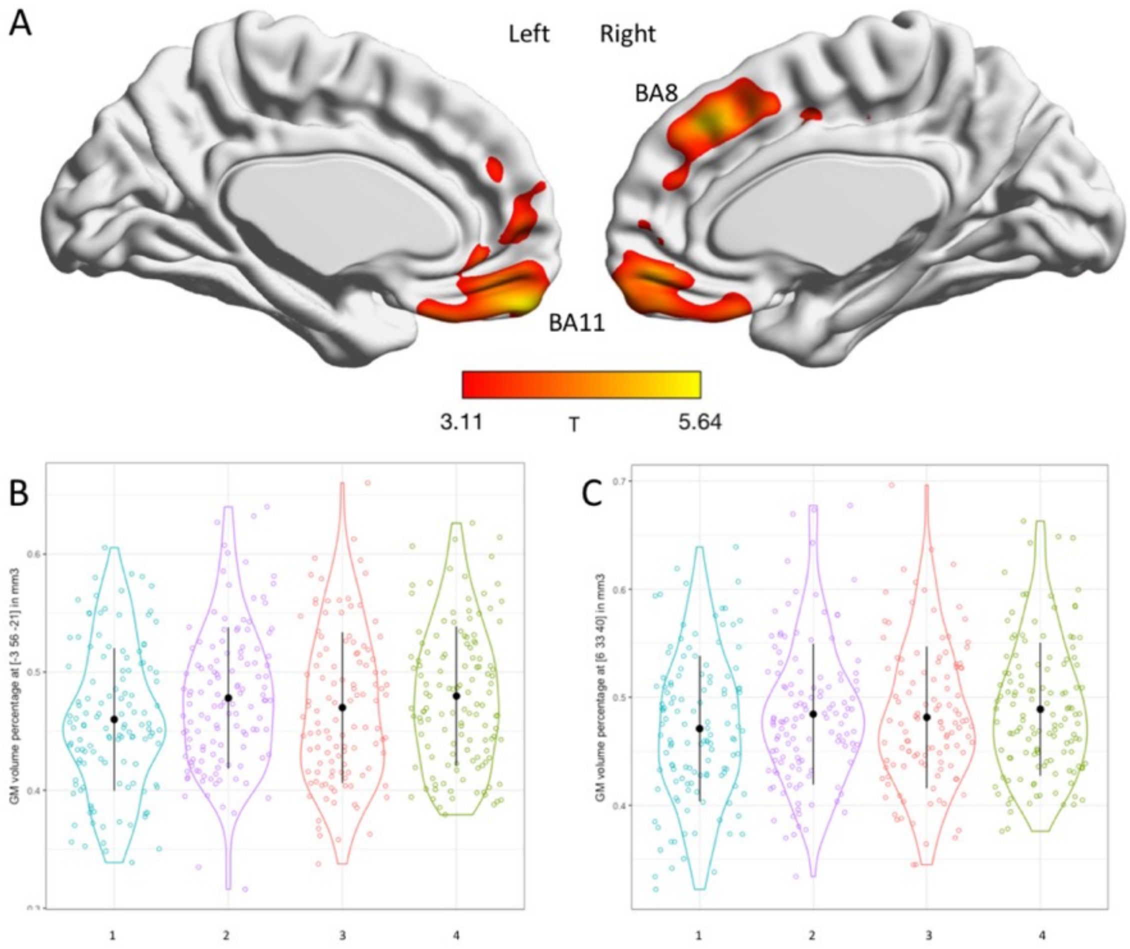

Fig. S6. ***Early-life urbanicity effect on grey matter volume correlates in MRI across four groups.*** The effect of early-life urbanicity on grey matter volume associated with MRI was tested in a general lineal model with early-life urbanicity (Group #1: individuals who were born in and continue to live in cities; #2: individuals who have lived in cities since before age 12; #3: individuals who were born in and continue to live in rural areas until age 12-18; #4: individuals lived in rural areas for >18 years since birth). (A) T-map of the rural > urban effects shown at $p<0.001$, although peaks survived $p<0.05$ voxel-wise whole-brain FWE-corrected. (B) Scatterplot of rural > urban findings at Brodmann Area 11 in medial prefrontal cortex (peak = [-3 56 -21], T = 5.64, cluster size = 556, $p<0.05$, voxel-wise whole-brain FWE-corrected). (C) Scatterplot of rural > urban findings at Brodmann Area 8 in medial prefrontal cortex (peak = [6 33 40], T = 5.48, cluster size = 198, $p<0.05$, voxel-wise whole-brain FWE-corrected).

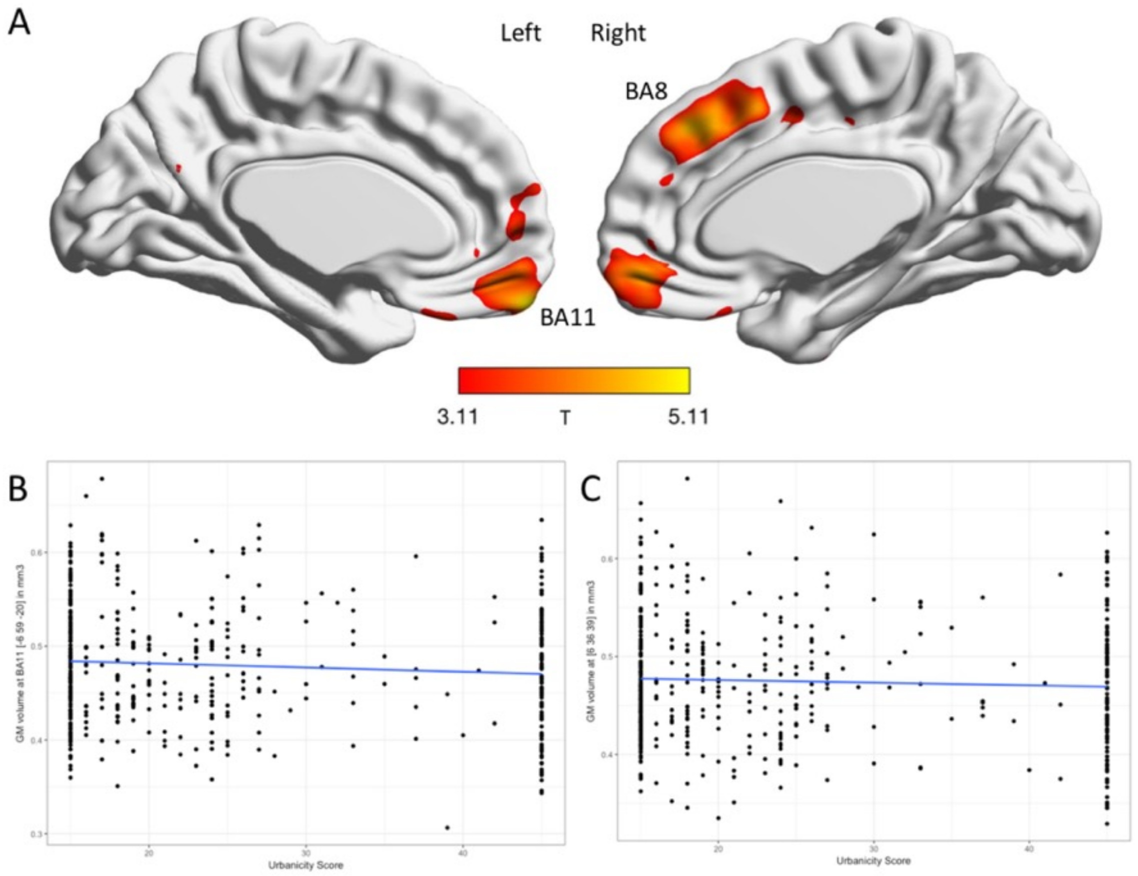

Fig. S7. ***Early-life urbanicity effect on grey matter volume correlates in MRI using the urbanicity score.*** The effect of early-life urbanicity on grey matter volume in MRI was tested in a correlation analysis with early-life urbanicity score ranging from 15 to 45. (A) T-map of the rural > urban effects shown at p<0.001 uncorrected but peaks survive p<0.05 voxel-wise whole-brain FWE corrected. (B) Scatterplot of rural > urban findings at Brodmann Area 11 in medial prefrontal cortex (peak = [-6 59 -20], T = 5.11, cluster size = 89, p<0.05, voxel-wise whole-brain FWE corrected). (C) Scatterplot of rural > urban findings at Brodmann Area 8 in medial prefrontal cortex (peak = [6 36 39], T = 4.67, cluster size = 20, p<0.05, voxel-wise whole-brain FWE corrected).

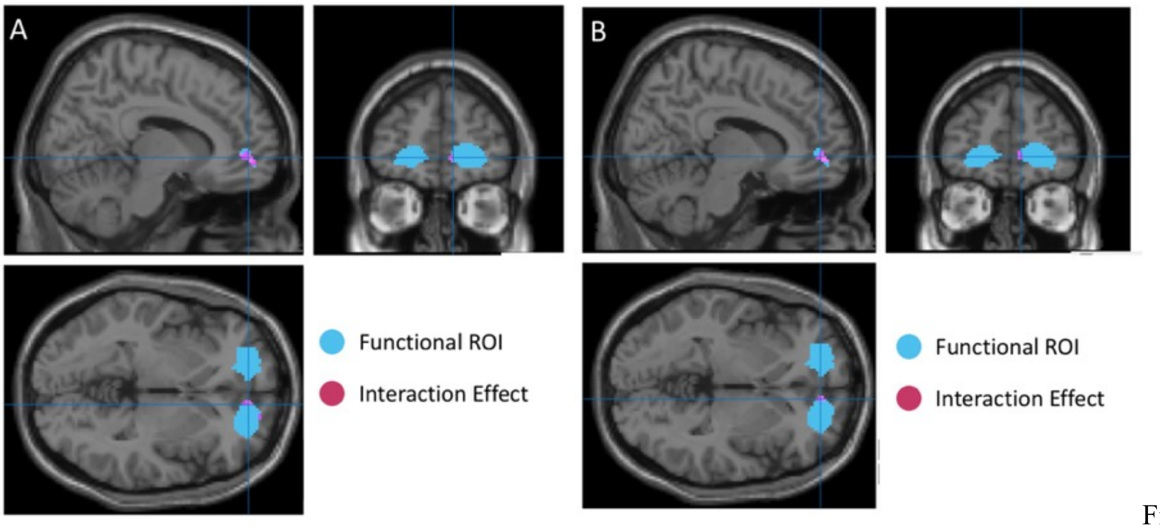

Fig.

Fig. S8. ***Interaction effects of trait anxiety-depression and two related definitions of urbanicity on medial prefrontal cortex engagement under interpersonal stress during working memory manipulation.*** (A) Interaction effects of trait anxiety-depression and urbanicity (four categories, as in Figure S2; N=394, x=8, y=54, z=-4, T=3.21; $p < 0.001$ uncorrected, $p<0.05$ FWE corrected within the mPFC ROI). (B) Interaction effects of trait anxiety-depression and urbanicity score (Urbanicity score, as in Figure S3; x=8, y=52, z=-2, T=3.19; $p < 0.001$ uncorrected, $p<0.05$ FWE corrected within the mPFC ROI).

Supplementary Tables:

Table S1. The effects of urbanicity, stress and task on behavioral data

Behavior	SS	df	MS	F	*P*
**Accuracy**					
Group (Urban/Rural)	0.002	1	0.002	0.159	0.690
Stress (Stress/Less-Stress)	0.393	1	0.393	60.597	< 0.001***
Task (WM Manipulation/Maintenance)	0.420	1	0.420	54.690	< 0.001***
Group * Stress	0.006	1	0.006	0.967	0.326
Group * Task	0.004	1	0.004	0.586	0.445
Task*Stress	0.300	1	0.300	46.672	< 0.001***
Group * Stress * Task	0.001	1	0.001	0.208	0.648
**Reaction Time**					
Group (Urban/Rural)	3.294	1	3.294	9.744	0.002
Stress (Stress/Less-Stress)	1.262	1	1.262	58.769	< 0.001***
Task (WM Manipulation/ Maintenance)	61.491	1	61.491	1662	< 0.001***
Group * Stress	0.007	1	0.007	0.341	0.560
Group * Task	0.030	1	0.030	0.785	0.376
Stress * Task	0.057	1	0.057	4.146	0.042
Group * Stress * Task	0.016	1	0.016	1.177	0.279

*** $P < 0.001$

Table S2. Brain activation of working memory maintenance and manipulation under social threat stress and less stress conditions (N=394, controlling for age, p< 0.05, voxel-wise whole-brain FWE corrected, cluster size > 100).

Peak Region	Cluster	x	y	z	T score
**Manipulation: Stress**					
L Middle Frontal Gyrus	112370	-32	50	16	18.74
R Middle Frontal Gyrus		44	36	24	25.30
R Cingulate Gyrus		6	16	44	41.27
L Middle Frontal Gyrus		-44	20	28	29.35
R Middle Frontal Gyrus		54	12	34	30.27
R Insula		32	24	0	34.69
L Insula		-34	18	2	33.14
L Insula		-40	-2	6	37.20
L Thalamus		-18	-10	-2	30.00
R Thalamus		14	-12	10	27.41
L Inferior Parietal Lobule		-44	-32	44	44.23
R Inferior Parietal Lobule		46	-36	42	33.00
L Hippocampus		-20	-30	-6	27.95
R Hippocampus		24	-26	-10	24.37
R Culmen		32	-54	-32	42.33
R Middle Occipital Gyrus		30	-68	32	33.09
L Precuneus		-24	-66	40	42.01
L Middle Occipital Gyrus		-28	-88	-2	37.57
R Occipital Lobe		28	-86	-6	37.60
**Manipulation: Less Stress**					
L Inferior Parietal Lobule	108004	-46	-34	44	44.11
R Cingulate Gyrus		6	16	42	43.83
L Middle Frontal Gyrus		-30	48	14	21.06
R Middle Frontal Gyrus		32	52	16	19.33
R Middle Frontal Gyrus		44	34	26	26.48
L Middle Frontal Gyrus		-44	20	26	31.29
R Inferior Frontal Gyrus		52	12	34	33.11
L Inferior Frontal Gyrus		-52	10	26	36.59
R Insula		32	24	-2	38.33
L Insula		-30	20	2	37.32
L Medial Frontal Gyrus		-2	6	50	42.42
L Middle Frontal Gyrus		-30	-4	56	41.90
R Middle Frontal Gyrus		28	0	58	31.43
L Lentiform Nucleus		-20	-6	0	32.68
R Lentiform Nucleus		16	-4	-2	28.63
L Insula		-40	-4	8	36.05

R Insula		42	0	6	27.88
L Thalamus		-12	-20	4	37.64
R Thalamus		12	-14	6	29.80
R Superior Parietal Lobule		26	-68	46	35.92
L Precuneus		-26	-64	42	43.71
L Middle Occipital Gyrus		-30	-88	-2	35.98
R Inferior Occipital Gyrus		28	-86	-8	35.68
**Maintenance: Stress**					
L Middle Frontal Gyrus	115766	-40	34	24	22.09
R Middle Frontal Gyrus		40	40	28	22.62
R Cingulate Gyrus		6	16	40	38.60
R Insula		34	20	2	29.90
L Insula		-32	18	4	29.60
L Insula		-40	-2	6	40.85
R Insula		46	4	2	30.92
L Thalamus		-12	-20	6	27.49
R Thalamus		6	-20	8	25.67
L Inferior Parietal Lobule		-44	-32	44	43.31
R Postcentral Gyrus		48	-30	40	26.36
L Precuneus		-18	-70	48	34.21
R Inferior Parietal Lobule		32	-58	48	31.64
L Middle Occipital Gyrus		-26	-88	-2	32.78
R Middle Occipital Gyrus		28	-86	-4	31.55
L Hippocampus		-18	-30	-8	23.30
**Maintenance: Less Stress**					
L Middle Frontal Gyrus	110896	-32	46	14	18.25
R Middle Frontal Gyrus		30	46	22	19.61
L Inferior Frontal Gyrus		-56	8	26	34.03
R Inferior Frontal Gyrus		54	10	36	30.39
Medial Frontal Gyrus		0	4	50	36.18
L Insula		-30	18	2	31.40
R Insula		32	20	2	29.56
L Insula		-40	-2	6	37.76
R Insula		40	0	6	29.47
L Thalamus		-12	-20	4	30.62
R Thalamus		14	-14	4	22.84
L Postcentral Gyrus		-38	-28	52	37.50
R Postcentral Gyrus		50	-24	38	25.86
L Precuneus		-26	-64	42	36.75
R Inferior Parietal Lobule		32	-58	48	30.93

L Middle Occipital Gyrus	-28	-88	-2	32.43
R Occipital Lobe	28	-86	-6	29.41

Table S3. Activation contrasts across working memory maintenance and manipulation under stress and less stressed conditions (N=394, controlling for age, p< 0.05, voxel-wise whole-brain FWE-corrected, cluster size > 100).

Peak Region	Cluster	x	y	z	T score
**Stress: Manipulation > Maintenance**					
R Insula	65620	32	26	0	19.54
L Insula		-30	26	0	19.61
L Middle Frontal Gyrus		-30	-2	56	18.26
L Medial Frontal Gyrus		-2	12	52	23.56
R Inferior Frontal Gyrus		46	8	26	19.56
R Middle Frontal Gyrus		28	-2	50	19.20
L Thalamus		-12	-14	4	14.5
R Thalamus		12	-16	8	11.07
L Hippocampus		-22	-32	-6	13.87
R Hippocampus		24	-28	-8	12.13
L Inferior Parietal Lobule		-46	-40	42	19.51
R Inferior Parietal Lobule		46	-38	42	19.65
R Superior Parietal Lobule		26	-66	44	28.04
L Precuneus		-24	-68	42	26.03
L Middle Occipital Gyrus		-30	-82	10	26.75
R Middle Occipital Gyrus		34	-82	2	28.22
**Less Stress: Manipulation > Maintenance**					
L Insula	64008	-30	26	0	20.29
R Insula		32	26	-2	19.93
L Inferior Frontal Gyrus		-44	6	28	26.71
R Inferior Frontal Gyrus		48	10	26	21.57
L Medial Frontal Gyrus		-4	12	50	25.33
L Thalamus		-12	-14	0	16.65
R Thalamus		12	-18	8	12.47
R Middle Frontal Gyrus		28	0	54	20.15
L Middle Frontal Gyrus		-28	-2	52	19.75
R Inferior Parietal Lobule		48	-38	46	22.61
L Inferior Parietal Lobule		-44	-42	42	20.72
L Precuneus		-24	-68	38	27.52
R Precuneus		28	-68	42	29.38
L Middle Occipital Gyrus		-30	-84	6	24.61
R Middle Occipital Gyrus		36	-82	2	28.54
**Stress: Maintenance > Manipulation**					
L Cingulate Gyrus	4666	-4	-42	34	15.60
R Cingulate Gyrus		10	-48	30	15.53

L Precuneus		-10	-64	22	10.88
L Angular Gyrus	1666	-46	-74	34	15.48
L Superior Temporal Gyrus		-56	-64	28	11.92
L Inferior Parietal Lobule		-66	-36	28	9.54
R Angular Gyrus	5762	52	-68	32	15.48
R Middle Temporal Gyrus		62	-40	-2	13.75
R Superior Temporal Gyrus		60	-58	16	13.52
R Inferior Parietal Lobule		62	-26	26	12.14
R Middle Temporal Gyrus		52	-8	-14	10.17
R Superior Frontal Gyrus	2168	14	50	36	8.85
R Middle Frontal Gyrus		24	30	46	8.04
L Medial Frontal Gyrus		-6	62	4	6.86
L Middle Temporal Gyrus	896	-58	-14	-14	8.25
L Insula		-42	-12	-6	7.90
L Middle Temporal Gyrus		-54	-2	-18	7.03
L Middle Frontal Gyrus	273	-24	26	42	6.89
L Superior Frontal Gyrus		-16	46	34	5.19
**Less Stress: Maintenance > Manipulation**					
R Cingulate Gyrus	4728	8	-50	28	16.84
L Cingulate Gyrus		-4	-44	32	15.52
L Precuneus		-10	-64	20	12.66
L Angular Gyrus	1793	-46	-74	34	15.12
L Superior Temporal Gyrus		-58	-62	22	9.56
L Supramarginal Gyrus		-60	-52	30	8.76
R Superior Temporal Gyrus	6236	62	-54	10	14.53
R Postcentral Gyrus		62	-26	22	13.77
R Middle Temporal Gyrus		60	-40	-2	13.21
R Superior Frontal Gyrus	3546	4	60	-6	10.86
R Superior Frontal Gyrus		14	50	38	9.60
R Medial Frontal Gyrus		8	56	14	8.56
L Insula	1121	-42	-12	-8	8.18
L Insula		-42	-18	-2	7.80
L Middle Temporal Gyrus		-62	-14	-14	7.55
R Inferior Frontal Gyrus	106	52	32	0	7.83

Table S4. Brain activation contrasts between stress and less stress conditions during working memory maintenance or manipulation (N=394, controlling for age, p< 0.05, voxel-wise whole-brain FWE-corrected, cluster size > 100).

Peak Region	Cluster	x	y	z	T score
**Manipulation: Less Stress > Stress**					
R Putamen	3627	18	14	-8	14.82
R Medial Frontal Lobe		20	52	-6	11.23
R Amygdala		28	-8	-12	11.19
L Extra-Nuclear	3657	-22	16	-10	14.67
L Putamen		-22	4	-6	13.05
L Caudate		-12	14	4	11.04
L Inferior Frontal Lobe	2531	-40	8	28	9.04
L Inferior Frontal Gyrus		-44	2	36	8.11
R Middle Frontal Gyrus	1667	46	12	30	8.85
R Middle Frontal Gyrus		54	26	32	7.57
R Middle Frontal Gyrus		44	30	22	6.45
R Inferior Parietal Lobule	1056	38	-62	46	8.79
R Midbrain	933	10	-18	-6	8.60
L Midbrain		-8	-18	-8	8.10
L Thalamus		-16	-22	0	6.84
L Middle Temporal Gyrus	285	-51	-32	-6	7.02
L Precuneus	538	-30	-64	38	6.89
**Maintenance: Less Stress > Stress**					
R Putamen	4075	18	14	-8	15.52
R Medial Frontal Lobe		18	52	-6	12.44
R Superior Temporal Gyrus		42	18	-36	8.52
L Putamen	4117	-20	14	-8	13.96
L Frontal Lobe		-18	50	-6	11.96
L Anterior Cingulate		-10	40	2	9.28
R Precentral Gyrus	840	58	-6	46	6.97
R Precentral Gyrus		54	-4	28	6.27
R Precentral Gyrus		52	-12	54	5.66
L Brain Stem	314	-10	-12	-10	6.87
L Middle Temporal Gyrus	416	-58	-30	-4	6.8
L Middle Temporal Gyrus		-52	-40	0	6.7
L Middle Temporal Gyrus		-56	-16	-8	4.93
L Middle Frontal Gyrus	1397	-42	0	44	6.72
L Middle Frontal Gyrus		-42	10	30	6.14
**Manipulation: Stress > Less Stress**					
R Fusiform	12046	34	-42	-4	12.54

L Temporal Lobe		-34	-46	-2	10.35
L Lateral Ventricle		-18	-44	14	9.70
R Lateral Ventricle		20	-42	14	11.34
R Extra-Nuclear		20	-2	26	8.17
L Extra-Nuclear		-18	-2	26	9.99
Inter-Hemispheric		0	-26	6	10.09
L Lateral Ventricle		-28	-52	10	11.21
R Lateral Ventricle		18	-40	12	11.47
L Precuneus	898	-10	-60	66	8.99
R Precuneus		6	-60	66	7.45
**Maintenance: Stress > Less Stress**					
L Extra-Nuclear	12370	-20	-4	26	10.16
R Extra-Nuclear		20	-6	26	9.28
L Extra-Nuclear		-22	-16	26	10.97
R Extra-Nuclear		26	-44	18	11.19
L Lateral Ventricle		-22	-36	12	10.20
L Para-Hippocampus		-34	-44	-4	11.12
R Lateral Ventricle		34	-42	-2	12.30
Inter-Hemispheric		0	-24	8	12.80
R Lateral Ventricle		20	-40	10	11.62
R Postcentral Gyrus	957	8	-58	68	8.13
R Precuneus		8	-74	54	7.76
R Precuneus		6	-68	60	7.47
L Inferior Parietal Lobule	105	-66	-30	30	5.96

Supplementary References:

1. Mortensen, P.B., *et al.* Effects of family history and place and season of birth on the risk of schizophrenia. *The New England journal of medicine* **340**, 603-608 (1999).
2. Lederbogen, F., *et al.* City living and urban upbringing affect neural social stress processing in humans. *Nature* **474**, 498-501 (2011).
3. Ashburner, J. VBM tutorial. in *Tech. repWellcome Trust Centre for Neuroimaging, London, UK* (Ashburner, John, 2010).
4. Weinberger, D.R. & Radulescu, E. Finding the Elusive Psychiatric "Lesion" With 21st-Century Neuroanatomy: A Note of Caution. *Am J Psychiatry* **173**, 27-33 (2016).
5. Tan, H.Y., *et al.* Catechol-O-methyltransferase Val158Met modulation of prefrontal-parietal-striatal brain systems during arithmetic and temporal transformations in working memory. *J Neurosci* **27**, 13393-13401 (2007).

6. Tan, H.Y., *et al.* Effective connectivity of AKT1-mediated dopaminergic working memory networks and pharmacogenetics of anti-dopaminergic treatment. *Brain* **135**, 1436-1445 (2012).
7. Wray, N.R., *et al.* Genome-wide association analyses identify 44 risk variants and refine the genetic architecture of major depression. *Nature Genetics* **50**, 668-681 (2018).
8. Purcell, S., *et al.* PLINK: a tool set for whole-genome association and population-based linkage analyses. *Am J Hum Genet* **81**, 559-575 (2007).
9. Howie, B.N., Donnelly, P. & Marchini, J. A flexible and accurate genotype imputation method for the next generation of genome-wide association studies. *PLoS genetics* **5**, e1000529 (2009).
10. Delaneau, O., Zagury, J.F. & Marchini, J. Improved whole-chromosome phasing for disease and population genetic studies. *Nature methods* **10**, 5-6 (2013).
11. Yengo, L., *et al.* Meta-analysis of genome-wide association studies for height and body mass index in approximately 700000 individuals of European ancestry. *Hum Mol Genet* **27**, 3641-3649 (2018).
12. Jansen, I.E., *et al.* Genome-wide meta-analysis identifies new loci and functional pathways influencing Alzheimer's disease risk. *Nat Genet* **51**, 404-413 (2019).
13. Arnsten, A.F.T. Stimulants: Therapeutic actions in ADHD. *Neuropsychopharmacology* **31**, 2376-2383 (2006).
14. Yoon, K.L., LeMoult, J., Hamedani, A. & McCabe, R. Working memory capacity and spontaneous emotion regulation in generalised anxiety disorder. *Cognition and Emotion* **32**, 215-221 (2018).
